# Development and validation of a 3D laryngeal model in surgical skills training

**DOI:** 10.1016/j.bjorl.2021.09.010

**Published:** 2021-11-15

**Authors:** Thiago Victal Saliba, Rui Sérgio Monteiro de Barros

**Affiliations:** Universidade do Estado do Pará (UEPA), Programa de Mestrado em Cirurgia e Pesquisa Experimental (CIPE), Belém, PA, Brazil

**Keywords:** Anatomical models, Endoscopy, Laryngology, Vocal folds, Microsurgery

## Abstract

**Objective:**

To present and validate a new simulation model for endoscopic phonomicrosurgery training as an improved teaching method.

**Methods:**

A low-cost artificial model was assembled using 3D printing, silicone, and gelatin. The study was designed to test the model's ability to carry out training and teaching of endoscopic phonomicrosurgery. The synthetic model was built to simulate normal and pathological vocal folds such as polyps, intracordal cysts and keratoses, made of silicone and gelatin and embedded in a larynx framework made by 3D printing. Simulations of endoscopic surgical procedures were performed and documented through photographs and videos and the images were submitted to the evaluation of a group of 17 otorhinolaryngologists who used a Likert scale questionnaire. The responses were submitted to an agreement analysis using the sum of the scores obtained for the responses as an appropriate level of validation. Cronbach's alpha index was calculated to measure the degree of the questionnaire internal consistency.

**Result:**

The evaluations indicated maximum approval for the model adequacy for use in practical classes and for the teaching of the procedures, as well as in the overall satisfaction with the model in the use of surgical training.

**Conclusion:**

The present proposal for training laryngeal endoscopic surgery in a 3D synthetic model is a viable option according to the validation methodology used in the present study.

## Highlights

• 3D model adequate for training in laryngeal endoscopic surgery.
• Simulation of different vocal fold lesions.
• Learning and teaching of laryngeal microsurgery in the laboratory.
• Surgical training with low cost, accessibility and replicability.
• New technologies for acquiring surgical skills.

## Introduction

One of the most frequent symptoms in the practice of otorhinolaryngologists is voice alteration (dysphonia), and its adequate evaluation is extremely important. Some types of dysphonia are caused by lesions that can or should be surgically treated, such as benign lesions of the vocal folds: cysts, polyps, nodules, Reinke's edema, sulci, among others,^1^ pre-malignant and malignant lesions,^2^ and neurogenic disorders. According to Jung et al.,^3^ the prevalence of benign vocal fold lesions in South Korea in 2015 was 12.47% and the incidence was 7.98%. According to Schultz,^4^ half of laryngeal cancer cases involve the vocal folds.

Surgical treatment by endoscopic access to the larynx (transoral approach) is usually the best option for benign lesions of the vocal folds and for voice improvement surgeries. However, Woo et al.,^5^ demonstrated that postoperative dysphonia can occur for multiple reasons, with scarring and rigidity in the vocal folds being the main causes of damage from phonosurgery,^6^, ^7^, ^8^ causing voice dysfunction due to the impairment in the vibratory characteristics of the lamina propria, and glottic closure impairment resulting from failures in mucosal coverage.

Endolaryngeal phonosurgery requires very specific surgical skills, which necessitate frequent training and practice. Traditionally, training in this procedure in medical residency services is performed during actual surgeries under the guidance of tutors. As these surgeries are sporadically performed, the learning curve for acquiring the skills is a long one. According to Shah et al.^9^ in a survey conducted with medical residents of the United States and Canada, 82% of them did not have the opportunity for training in phonosurgery in laboratories and 87.4% of them reported that they would be more confident in performing the surgery if they had a greater opportunity for training in models. Only 18.8% said they were very satisfied with the acquired experience.

Training in experimental models of laryngeal endoscopic surgery can make up for this deficiency, allowing the practice in a controlled environment, without affecting patient safety due to possible errors made during an actual surgery. They are also excellent methods to evaluate the performance of those carrying out the simulation.^9^, ^10^, ^11^, ^12^, ^13^, ^14^, ^15^, ^16^, ^17^, ^18^, ^19^

Human cadaver larynx,^10^ animal larynx^18^, ^20^ and artificial larynx models are commonly used. Synthetic models have, as a disadvantage compared to natural models, the difficulty in replicating the histological structure of the vocal folds, which have rigidity, tension and elasticity characteristics that constantly vary during their oscillation and vibration, resulting from tissue movement (muscle, connective tissue and mucosal coverage) and the air flow that hits them.^21^

Vocal fold biomechanical studies have been carried out, mainly using Young's modulus of elasticity – a mechanical property that measures the stiffness of a material when it is submitted to external traction or compression. Alipour and Vigmostad,^21^ observed that the behavior of the elastic properties is not linear, oscillating with an increase of 10–15 times between the values ​​found in the measurements made at low and high stress strain in Young's modulus. The mean measure of low strain in Young's modulus was 30 kPa in the longitudinal direction and 1 kPa in the transverse direction. Kelleher et al.^22^ described values ​​of 14 kPa for the vocal ligament and 39 kPa for the vocal fold cover (epithelium and superficial layer of the lamina propria). These data provide a general range of measurements for Young's modulus and would be a starting point for the creation of a synthetic vocal fold. However, this non-linear behavior increases the difficulty in finding a suitable synthetic material to replicate such vocal fold characteristics. With technological evolution and continued research, the trend is to expand the availability and development of new materials^23^, ^24^, ^25^ that can achieve this goal.

This study reports the development and further validation of a synthetic model for training in endolaryngeal surgery that allows simulating some of the most common diseases of the vocal folds, using accessible and low-cost materials, which, even though they do not fully replicate the viscoelastic properties of the vocal folds, are able to represent them regarding adequate size and consistency.

## Methods

To validate the synthetic laryngeal model as a tool for surgical training, a simulation station for laryngeal microsurgery was set up using a surgical microscope (DFV®, MC-M31) with 400 mm objective lens coupled to a video camera (Toshiba® IK-CU44a) with a capture board (Dazzle®) connected to a notebook computer (Dell®) to carry out image recording. A set of laryngeal microsurgery instruments containing rigid laryngoscope, triangular fenestrated grasping forceps, straight microscissors and those curved to the right and left, straight and curved alligator grasping forceps, stylus blade, lancet, and retractor were used. As support for the laryngoscope, an artifact was made of PVC pipes as described by Zambricki et al.^13^ was used and, to fix the synthetic model of the larynx, a support made with a polyethylene board and nylon clamps, constructed by the author, was utilized.

### Steps of making and assembling the synthetic model

The synthetic model features a laryngeal structure made of polyamide by 3D printing and vocal folds made of gelatin and silicone.

#### Laryngeal structure

**Figure 1:**
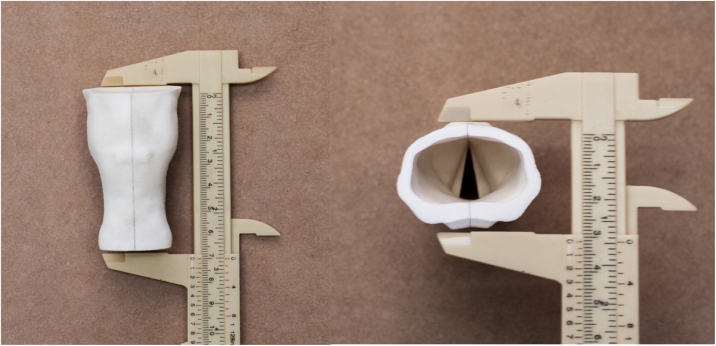
(Left) Images of the anterior aspect of the model measured in centimeters. (Right) Image of the superior plane of the larynx printed in 3D, measured in centimeters. Source: the author.

**Figure 2:**
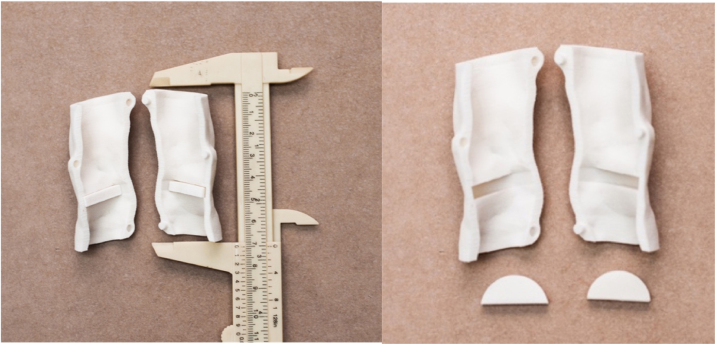
(Left) Internal image of the larynx after midline uncoupling. (Right) Internal image of the larynx after removal of the vocal folds. Note the recesses for fitting the vocal folds. Source: the author.

It was constructed with a shape and dimensions similar to the human larynx. It has a sagittal division into two hemilarynges, which are joined medially, allowing coupling and uncoupling. The lateral recesses in the inner part of the hemilarynges were designed to house artificial vocal fold parts, which are fitted, and allow multiple exchanges of the vocal fold parts and reuse of the structure (Figure 1, Figure 2).

The structure construction method was through 3D printing, being developed in partnership with the Renato Archer Information Technology Center.

During the development process, images acquired by Computed Tomography (CT) of the neck in extension were collected using Digital Imaging and Communications in Medicine (DICOM), with a slice thickness of 1.25 mm. Files in the Standard Template Library (STL) extension were used for the production of the 3D printed models.

For the segmentation of images from the CT, the InVesalius software was used, which stacked the 2D images in DICOM format and generated a 3D volumetric mesh with STL extension.

Then, two software were used to perform the modeling: Mashmixer® to keep the anatomical proportions of the glottic region uniform with the rest of the model and Magics®, which allowed the construction of the structure as two hemilarynges (divided longitudinally in the sagittal plane), and the recesses for fitting the vocal folds.

The 3D printing process performed for this model was the SLS (Selective Laser Sintering) and the material used by SLS was polyamide, a polymer made of nylon in powder form.

#### Vocal folds

The vocal folds were built as a peripheral extension to allow their insertion into the internal recesses of the laryngeal structure and its exchange after the training sessions. Artificial vocal folds of silicone and gelatin were produced through modeling using molds made of polyamide through 3D printing and with silicone rubber (Poli MX23, Polisil®), simulating vocal folds without pathological alterations and vocal folds with polyps.

**Figure 3:**
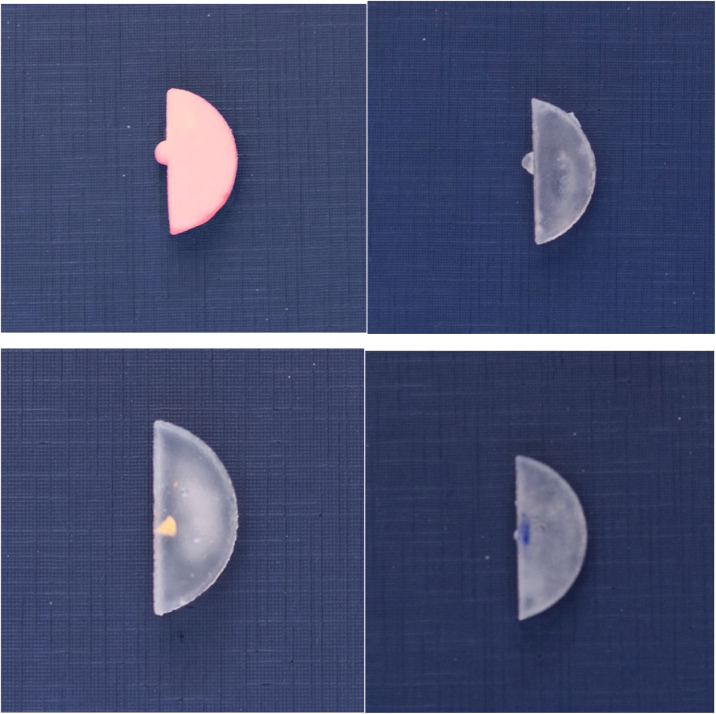
Synthetic silicone and gelatin vocal folds. Source: the author.

The silicone vocal folds (Poli MX23, Polisil®) were modeled reproducing polyps. The gelatin vocal folds (Dr. Oetker® colorless gelatin) were modeled reproducing three types of lesions: polyp, cyst and keratosis. Gelatin vocal folds with polyps were manufactured in a silicone mold shaped as the vocal fold with polyp. Vocal folds simulating cysts were produced by placing small, yellow-pigmented silicone spheres, measuring approximately 1–2 mm, over the free edges of the gelatin folds. Vocal folds simulating keratosis were produced by coloring the surface of the gelatin piece using a blue pen with a 4 mm porous tip (Figure 3).

#### Model assembly

Once the construction of the structure and the vocal folds was completed, the vocal fold that simulated the desired lesion was fitted into the projected recess of the laryngeal structure; the two hemilarynges were coupled, the model was positioned on the larynx support and the simulation started.

### Simulation of surgeries

**Figure 4:**
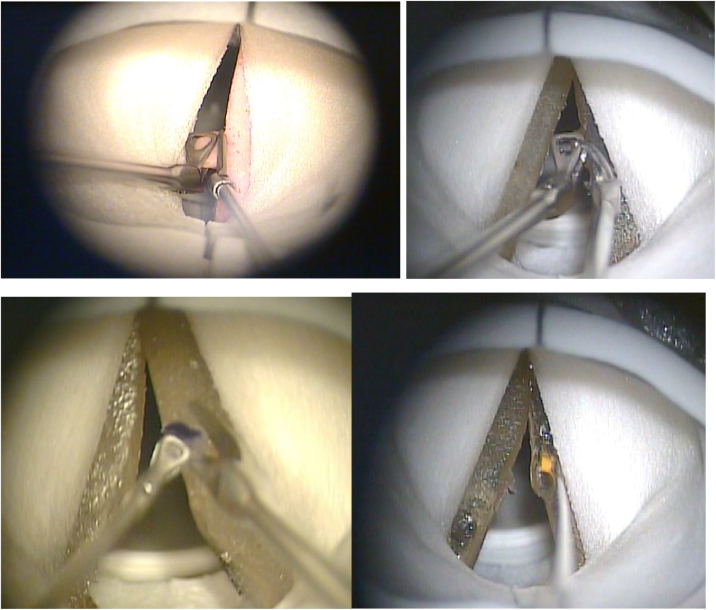
Microscopic image of surgical simulations in the larynx model. Source: the author.

Four surgical procedures were simulated in the laryngeal model by the author of the study: silicone vocal fold polyp excision, gelatin vocal fold polyp excision, gelatin vocal fold cyst removal, and decortication of keratosis in gelatin vocal fold (Fig. 4). For an adequate analysis of the model size, a ruler with measurements in centimeters was available (Figure 1, Figure 2). Simulation documentation was carried out through video images (in this article documented only by video capture image in Fig. 4).

### Validation

A group of 17 otorhinolaryngologists with at least 3 years of experience participated in this study as evaluator judges through voluntary recruitment using the snowball sampling technique in March 2021, after the project was approved by the Research Ethics Committee of Universidade do Estado do Pará-UEPA, campus VIII, under Opinion N. 4.604.547 (CAAE: 42691021.3.0000.8607), and signature of the free and informed consent form.

The participants answered a demographic data questionnaire (Table 1) prior to the analysis of the simulations. Afterwards, they analyzed the images of the model and simulations, and answered another questionnaire containing 9 statements (Table 2), with response options according to a 5-point Likert scale, showing the degree of agreement regarding the characteristics of the model on anatomical references, representation of pathologies and degree of likelihood of surgical simulation. An overall score for the model was also requested, with a value ranging from 1 to 10.

**Table 1:** Profile of the model evaluators, UEPA, 2021.

Profile of the evaluators	Frequency	% (n = 17)
Gender		
Female	6	35.3%
Male	11	64.7%
Age range (years)		
<30	1	5.9%
30 to 39	5	29.4%
40 to 49	7	41.2%
≥50	4	23.5%
Minimum/Mean/Maximum	28/42.2/55	
N. of endoscopic laryngeal surgeries		
Less than 50	9	53.0%
Between 50 and 100	3	17.6%
More than 100	5	29.4%
Professional performance time (years)		
<10	4	23.5%
10 to 19	7	41.2%
≥20	6	35.3%
Minimum/Mean/Maximum	03/14.8/30	

Source: Applied protocol.

**Table 2:** Final score of the sum of the answers to the statements after the model assessment, UEPA, 2021.

Statement	Average score received (max. = 5)	% achieved
The vocal folds seem to have adequate consistency during manipulation	4.11	82.4%
The surgical simulation in the model shows an adequate level of difficulty - the surgical technique applied is similar to reality	4.23	84.7%
The model adequately replicates vocal fold injuries - polyp, cyst, keratosis	4.23	84.7%
The model reproduces the real conditions of an endolaryngeal surgery	4.23	84.7%
The model simulates the reality	4.29	85.9%
The model has the anatomical references (vocal folds, glottic space) necessary to perform endolaryngeal surgery	4.76	95.3%
The training in this model will improve performance in an actual surgery.	4.94	98.8%
The model, as a whole, is satisfactory for surgical training.	5	100.0%
The model is adequate for use in practical classes and for the teaching of the procedure.	5	100.0%
Average of overall evaluation (max = 45)	40.82	90.7%
Score of the model (Min./Mean/Max.)	7.0/8.9/10.0	

Source: Applied protocol.

The following score was used as an evaluation metric for the 9 statements: Strongly Disagree = 1; Partially Disagree = 2; Neither Agree, nor Disagree = 3; Partially Agree = 4; Strongly Agree = 5.

The final score was obtained using the average of the sum of the scores given by the 17 participants to the 9 statements. According to the method, a score from 9 to 18 is considered insufficient and not validated; from 19 to 27, it is regular and not validated; from 28 to 36, it is good and validated with reservations (requiring improvements for a new validation process); and from 37 to 45, it is excellent and validated for use as a training tool.

To measure the degree of internal consistency of the applied questionnaire, the Cronbach's alpha index was calculated,^26^ and a value of 0.70 was considered the minimum acceptable value.

## Results

The evaluator judges were mostly male individuals (64.7%), with age ranging from 28 to 55 years, with an arithmetic mean of 42.2 years. The age group with the highest proportion was professionals aged between 40 and 49 years (41.2%), as shown in Table 1.

Regarding the professional profile, the time of professional experience ranged between 3 and 30 years, with an arithmetic mean of 14.8 years, as shown in Table 1.

The evaluations showed maximum approval for the model adequacy to be used in practical classes aiming to teach the procedure, as well as overall satisfaction with the model for simulation in surgical training (100% approval each). The second highest approval was the statement saying that training with the assessed model will improve performance in an actual surgery (98.8%).

**Figure 5:**
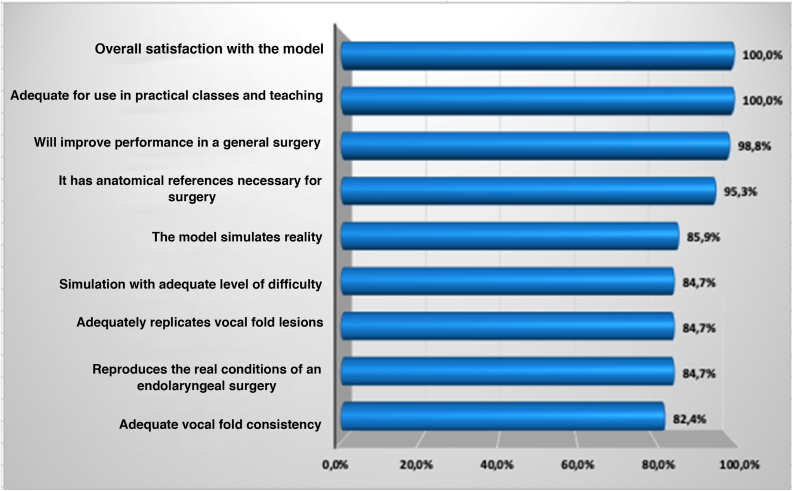
Final score of the sum of the answers to the statements after the evaluation of the model, UEPA, 2021. Source: Applied protocol.

As for the other questions, the method showed the following results: general anatomical references obtained 95.3% of approval; the model simulates reality obtained 85.9% of approval; adequate level of difficulty, anatomical references in relation to the vocal folds, and reproduction of the actual surgical conditions obtained 84.7% of approval; adequate consistency of the vocal folds obtained 82.4% of approval (Table 2; Fig. 5).

All statements obtained approval >82.0%. The lowest score was obtained by the statement that deals with the adequate consistency of the vocal folds during manipulation, reaching 82.4% of approval.

In the overall score given to the model by the evaluators, the minimum was 7.0 (seven) and the maximum was 10.0 (ten), with an arithmetic mean of 8.9, as shown in Table 2.

The calculation of Cronbach's Alpha index reached a value of 0.853; with the value of 0.70 being the minimum acceptable.

## Discussion

Study and training in surgery using simulation models allow the acquisition of essential skills for professional practice in any surgical discipline, as stated by Martin et al.,^27^ and it is also an essential condition in otorhinolaryngology.^28^ The need to use models for training and teaching laryngeal endoscopic surgery is widely recognized, as elucidated by Dailey et al.,^10^ Contag et al.^12^ and Shah et al.^9^ They are essential for learning and training, allowing the surgeon to improve their skills and confidence, resulting in surgical error reduction and better results.

Given the continuous demand for simulator-based training, some endolaryngeal surgery models have been described. As demonstrated by Javia et al.^29^ unlike otologic and sinonasal surgery models, phonosurgery models are not yet relevantly present in dissection and surgical simulation laboratories.

The ideal model must be realistic, accessible and low-cost. The human cadaver larynx has limited supply, demands technical criteria for storage and handling, and is expensive. Animal larynges, albeit low cost (in the case of a pig’s larynx) and better supply access, also show difficulties in storage and handling. Hence, we agree with Holliday et al.^30^ stating that this is a significant disadvantage of these models, despite their anatomical superiority. Synthetic models might not be as realistic as animal ones, but after the difficulty of the initial design and construction phase, they are replicable, easy to store, and can be low cost, as already explained by Contag et al.,^12^ Zambricki et al.,^13^ and Holliday et al.^30^

The present article presents and validates a new synthetic model, which, given the technical difficulty of replicating the viscoelastic properties of the vocal folds, was developed aiming to reproduce the dimensions and texture of the human vocal folds, and their respective lesions, in an attempt to provide the best possible perception of a surgical simulation. The average cost, of which price was based on the calculation of the unit value per gram of the used materials, not including the equipment, was R$ 5.70 for the laryngeal structure, R$ 0.04 for the silicone vocal fold and R $0.15 for the gelatin vocal fold. Our model, as well as that of Contag et al.,^12^ Zambrick et al.,^13^ and Holliday et al.,^30^ shows low cost, accessibility and replicability. Despite the initial difficulty in printing the 3D structure, it has the advantage of disclosing the necessary anatomical references to perform endolaryngeal surgery (95.3% of the maximum score) and adequate consistency of the vocal folds (82.4% of the score). Another similar synthetic model, made by Klein et al.,^14^ has very realistic qualities, including three-layer artificial vocal folds, but it is expensive and does not allow the self-sufficient construction, like our model. There are few synthetic models in 3D printing that are currently available. Lee et al.^31^ built a model for percutaneous injection laryngoplasty training that does not include endoscopic training. Kavanagh et al.^32^ developed a pediatric laryngeal model with excellent anatomical quality that replicates only pediatric pathologies.

As well as several previous publications referring to synthetic models, our results demonstrated that this model has the capacity to improve surgical training and can be used in practical classes.

One disadvantage of our study is the small sample size, the visual appreciation only of the surgical simulations in the model, and the absence of a methodology for quantitative assessment of skills acquisition after surgical training using the model. It also did not consider the difference in assessment between novice and expert professionals. The lack of the multiple layers of the artificial vocal folds is another limitation that must be overcome.

This training model is another tool that can help the otorhinolaryngologist’s performance and improve surgical results. The results encourage more commitment to this type of research, also serving as an incentive for other researchers to create new realistic training models.

## Conclusion

The present proposal for training in laryngeal endoscopic surgery in a 3D synthetic model is a viable learning and teaching option according to the validation methodology used in this study.

## Funding

A Patent Deposit Application was made at INPI, case number BR 1020210061634 by UEPA, with the authors declared as inventors.

## Conflicts of interest

The authors declare no conflicts of interest.
